# The role of N-myristoyltransferase 1 in tumour development

**DOI:** 10.1080/07853890.2023.2193425

**Published:** 2023-05-04

**Authors:** Hong Wang, Xin Xu, Jiayi Wang, Yongxia Qiao

**Affiliations:** a School of Public Health, Shanghai Jiao Tong University School of Medicine, Shanghai, China; b Department of Clinical Laboratory, Shanghai Chest Hospital, Shanghai Jiao Tong University School of Medicine, Shanghai, China; c Shanghai Institute of Thoracic OncologyShanghai Chest Hospital, Shanghai Jiao Tong University School of Medicine, Shanghai, China; d College of Medical Technology, Shanghai Jiao Tong University School of Medicine, Shanghai, China; e The International Peace Maternity and Child Health Hospital, School of Medicine, Shanghai Jiao Tong University, Shanghai, China

**Keywords:** N-myristoyltransferase 1, myristoylation, tumour, NMT1 inhibitor

## Abstract

N-myristoyltransferase 1 (NMT1) is an indispensable eukaryotic enzyme that catalyses the transfer of myristoyl groups to the amino acid terminal residues of numerous proteins. This catalytic process is required for the growth and development of many eukaryotes and viruses. Elevated expression and activity of NMT1 is observed to varying degrees in a variety of tumour types (e.g. colon, lung and breast tumours). Furthermore, an elevated level of NMT1 in tumours is associated with poor survival. Therefore, a relationship exists between NMT1 and tumours. In this review, we discuss the underlying mechanisms by which NMT1 is associated with tumour development from the perspective of oncogene signalling, involvement in cellular metabolism, and endoplasmic reticulum stress. Several NMT inhibitors used in cancer treatment are introduced. The review will provide some directions for future research.

**Key Messages**
- Elevated expression and activity of NMT1 is observed to varying degrees in a variety of tumour types which creates the possibility of targeting NMT1 in tumours.
- NMT1-mediated myristoylation plays a pivotal role in cancer cell metabolism and may be particularly relevant to cancer metastasis and drug resistance. These insights can be used to direct potential therapeutic avenues for NMT1 inhibitors.

## Introduction

Tumourigenesis is characterized by biological properties such as sustained proliferation, resistance to apoptosis, metastasis, epithelial mesenchymal transition, metabolic reprogramming and immune escape [1], and is caused by altered activity of intracellular signalling, metabolic and gene regulatory networks. Protein post-translational modifications are tightly associated with in these alterations [2–4]. Protein post-translational modifications are covalent attachments of specific motifs to amino acid residues of proteins under the catalytic action of enzymes. Typical post-translational modifications are methylation, phosphorylation, ubiquitination and lipidation [4]. In recent years, the importance of one of these lipid modifications, myristoylation, in the development of human tumourigenesis has emerged [5,6]. A series of studies has shown that myristoylation plays an essential role in signal transduction, protein stability and protein localization at the membrane [7].

Myristoylation is the irreversible covalent bonding of myristic acid (also known as tetradecanoic acid, a 14-carbon saturated fatty acid) to the N-terminal glycine of a protein with myristoyl coenzyme A as the donor [8]. Recent studies have revealed that lysines can also be myristoylated [9]. In eukaryotes, myristoylation occurs in 0.5–3% of the cellular proteome [10]. Although myristoylation affects only a minority of eukaryotic proteins, it is vital for the survival and development of organisms and has implications for various diseases such as cancer [6] and malaria [11]. Myristoylation controls protein function by targeting proteins to specific locations, promoting specific protein–protein and protein–lipid interactions, and causing ligand-induced conformational changes [12]. Familiar myristoylated proteins include the β subunit of calmodulin independent protein phosphatase, the myristoylated alanine-rich C kinase substrate, the α subunit of several G proteins, and several ARF proteins involved in ADP ribosylation [13,14].

Originally, proteins were postulated to undergo co-translational myristoylation only after the initiating methionine residues were removed by methionine aminopeptidase. Subsequent studies found that post-translational myristoylation modifications also occur when cryptic internal glycine residues were exposed through the action of caspases in apoptotic cells [15,16]. New substrates and functions for myristoylation have also been uncovered recently, such as the myristoylation of EZH2. Myristoylation of this protein is associated with liquid–liquid phase separation in tumour growth [17], which uncovers a role for myristoylation in tumour development. This observation suggests that new substrates and functions of myristoylation remain unidentified [18,19], and reveals a broad prospect for future development in the field of myristoylation.

## Description of NMT1

Myristoylation plays a role in signal transduction, immune regulation and tumour development [7,20–23]. The process of myristoylation relies on a key enzyme, N-myristoyltransferase (NMT). During myristoylation, myristoyl coenzyme A binds to NMT and then the peptide substrate, and finally myristoyl coenzyme A is transferred to the N-terminal glycine residue of the substrate and NMT releases coenzyme A molecules and myristoylated proteins [8,10]. NMT is an indispensable enzyme for the growth and development of many eukaryotes and viruses [18,24–27] and is usually present *in vivo* as an isoenzyme [21]. Two major isoforms, N-myristoyltransferase 1 (NMT1) and N-myristoyltransferase 2 (NMT2), exist in higher eukaryotes and they share approximately 77% amino acid sequence similarity [28]. In humans, NMT2 exists as a single 65 kDa protein, while NMT1 exists as four different isoforms ranging in size from 49 to 68 kDa [28].

NMT1 and NMT2 have unique substrate affinities and differ considerably in function. Yang et al. [26] found that NMT1 knockout mice undergo death during embryogenesis and Ducker et al. [29] found that interfering with NMT1 but not NMT2 expression inhibited cancer cell proliferation in an *in vitro* model. Suppression of NMT2 indicated a potent role for this isoform in apoptosis [29]. The indispensable role of NMT1 in embryonic development and cell proliferation indicates the importance of research concerning this enzyme.

Although the field of myristoylation and NMT1 is new, more attention is warranted because of the close connection between NMT1 and tumourigenesis. Elevated expression and activity of NMT1 are observed to varying degrees in a variety of tumours (such as colon, lung and breast tumours) [6,30]. In addition, poor survival outcomes of tumours are associated with NMT1 levels in the tumours [31]. Growing evidence suggests that NMT1 regulates cancer cell metabolism [32]. Furthermore, NMT inhibitors show promise as potential therapeutic agents in haematological malignancies [33]. This review provides an overview of recent advances concerning the relationship between NMT1 and various tumours, thus highlighting the unique role of NMT1 in tumours and providing suggestions for future research areas.

## The relationship between NMT1 expression and tumours

### Solid tumours

Elevated NMT expression and activity is observed in a variety of solid tumours. The direct involvement of NMT in tumourigenesis was first observed in colon cancer, and NMT expression and activity is directly related to colon cancer progression [34–36]. NMT activity is significantly higher in rat colon tumours compared with normal rat colon mucosa. Furthermore, patients with colon cancer have altered NMT expression and localization in the peripheral blood and bone marrow [37]. These findings suggest that NMT could be used as a diagnostic or prognostic tool or a blood marker for colon cancer. In addition, significantly elevated NMT1 mRNA expression has been observed in lung cancer, and expression usually increases with disease progression, with NMT1 expression being 3.5-fold higher in patients with lung cancer than healthy individuals by stage IV [30]. In breast epithelial cells, proliferative capacity correlates with NMT activity [38], and breast cancer patients have elevated NMT1 expression in tissue microarrays [39]. Therefore, NMT1 is a potential diagnostic biomarker for breast cancer.

There is a correlation between poor survival outcomes and high NMT1 levels in tumours. In breast cancer, elevated NMT1 protein levels are associated with high overall histological grade, high Ki67 and low hormone receptor expression [31]. Furthermore, higher NMT1 mRNA levels are associated with poorer patient prognosis in ovarian cancer [40]. NMT1 is also associated with the development of other cancers, such as liver cancer [41], oral cavity cancer [42], brain tumours [43] and gallbladder cancer [44], but the underlying mechanisms are still unclear.

### Hematologic tumours

Although aberrant expression of NMT1 had not been reported in hematologic tumours, the findings of a drug trial implicated NMT1 in hematologic tumour development. A sensitivity trial for a novel NMT inhibitor called PCLX-001 involving 300 cancer cell lines from all major cancer types found that the inhibitor had a dramatic inhibitory effect on the growth of hematologic malignancies [33]. The researchers proposed that the greater susceptibility of haematological cancer cells than other cancer cells to PCLX-001 may result from altered expression of NMT1 or NMT2. Subsequently, NMT1 activity has been confirmed as being essential for lymphoma cell survival in a genome-wide Crispr-Cas9 assay, strengthening the evidence for a role of NMT1 in hematologic tumours [45].

## Mechanisms by which NMT1 affects tumours

**Figure 1.**
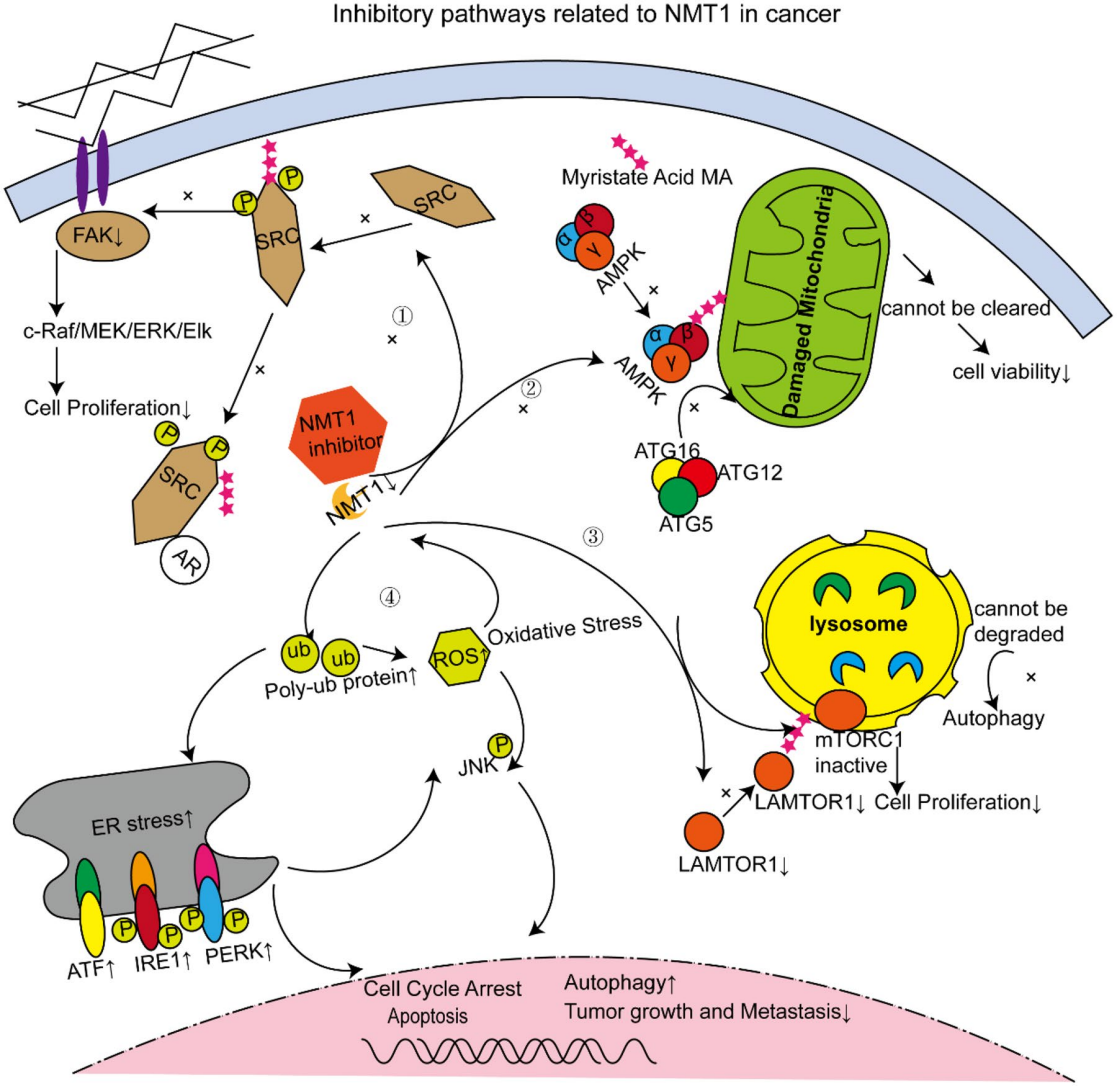
Signalling pathways associated with inhibition of N-myristoyltransferase 1 (NMT1) in cancer cells. ① NMT1 inhibition results in aberrant signalling of the Src oncogene and disrupts its scaffolding function for binding to the androgen receptor. ② NMT1 inhibition affects the aggregation of adenosine 5′-monophosphate (AMP)-activated protein kinase β (AMPKβ) activation into damaged mitochondria and the binding of the autophagy-related genes 16 (ATG16) complex to mitochondria. ③ NMT1 inhibition causes a decrease in overall late endosome/lysosomal adaptor, MAPK and mTOR activator 1 (LAMTOR1) protein levels and a downregulation of LAMTOR1 myristoylation such that the protein fails to localize to the lysosomal membrane, thereby preventing mTOR complex 1 (mTORC1) activation while impairing lysosomal self-degradation and leading to autophagic flux blockage. ④ NMT1 inhibition causes endoplasmic reticulum stress and oxidative stress, resulting in cell cycle arrest and apoptosis, along with activation of autophagy.

The effects of NMT1 on cancer development are extensive, especially in colon, lung, breast and ovarian cancers, and involve proto-oncogene tyrosine-protein kinase Src signalling and mechanisms of cellular metabolism (mitochondria, lysosomes), endoplasmic reticulum (ER) stress and lipid recoding (Figure 1).

### Oncogene Src activation

NMT1-mediated myristoylation leads to aberrant oncogene Src signalling, conferring cancer cells with greater aggressiveness and proliferative capacity, thereby promoting cancer development. The *Src* gene, the first proto-oncogene identified, expresses the protein product c-Src, a membrane-bound non-receptor tyrosine kinase, which is a key factor in many extracellular and intracellular signalling cascades [46]. In colon cancer cell lines, elevated NMT expression is accompanied by increased concentrations of Src [47]. Blocking c-Src myristoylation modification in colon cell lines reduces colony formation and slows proliferation [48] and non-myristoylated c-Src has decreased kinase activity [49]. Ducker et al. [29] further demonstrated that cell proliferation slowed upon NMT1 inhibition because NMT1 is responsible for myristoylation of Src. Blocking Src myristoylation leads to loss of focal adhesion kinase activity and the consequent downregulation of the c-Raf/MEK/ERK pathway [29] (Figure 1, ①).

Suppression of NMT1 was found to induce cell cycle arrest, proliferation inhibition and malignant growth inhibition in prostate cancer [50]. Myristoylation is involved in protein folding and promotes Src kinase to switch to its active conformation, leading to tyrosine phosphorylation (detected by pSrc(Y416)) [51] and downstream signalling, which is involved in cell proliferation. In prostate cancer, inhibition of NMT1 also blocks the scaffold function of Src kinase and prevents protein–protein interaction with the androgen receptor (AR), thus inhibiting androgen-independent AR activation (Figure 1, ①). Therefore, inhibition of NMT1 prevents the myristoylation of Src, which can eliminate the tumourigenic capacity of Src, while also interrupting the synergistic effect of Src with AR in mediating tumour invasion.

Compared with the numerous Src kinase inhibitors that target only the ATP binding site [52], inhibition of NMT1 provides an additional pathway to intervene in Src oncogenic signalling. Given the elevated expression and activity of Src in prostate and colon cancers, targeting the NMT1–Src axis provides a novel approach to inhibit tumour progression, particularly in Src-driven tumours.

### Cellular metabolism

Cancer metabolism is highly dynamic and complex, as disruption of a single branch of the metabolic process can alter cellular metabolism as a whole. Therefore, there is a growing interest in impairing or completely inhibiting metabolic pathways that are elevated in cancer cells by inhibiting the enzymatic activity of relevant proteins. Targeting cancer metabolism provides alternative ideas for improving widely applicable drugs. Among them, adenosine 5′-monophosphate (AMP)-activated protein kinase (AMPK) and mTOR are key molecules in the regulation of intracellular metabolism and important molecules related to NMT1 in lung cancer.

Studies in lung and breast cancer cell lines have shown that the AMPK complex attaches to damaged mitochondria, and that the attachment of AMPK to damaged mitochondria induces the linkage of AMPK to autophagy-related genes (ATG) 16-ATG5-12 and mediates the recruitment of the ATG16 complex to the site of damage (Figure 1, ②). This series of processes relies on NMT1-mediated myristoylation of the AMPKβ subunit [40]. Although both NMT1 and NMT2 are expressed in lung cancer cells, AMPK is only specifically regulated by NMT1. Therefore, NMT1 has an essential part to play in AMPK-dependent monitoring of mitochondrial damage and the induction of mitochondrial autophagy. Monitoring of damaged mitochondria helps to maintain cancer cell viability through the intrinsic apoptotic pathway; therefore, blocking effective mitochondrial monitoring in cancer cells by blocking AMPK myristoylation might reduce cancer cell viability. This mechanism represents a potential target for cancer therapy.

Lysosomes are complex multifunctional organelles with important roles in cellular metabolism [53]. Lysosomes are capable of mediating the enzymatic degradation of vesicular cargo and also serve as a functional platform for mTOR complex 1 (mTORC1) activation [54]. NMT1 is required for lysosomal function in cancer cells. In lung, breast and ovarian cancer cell lines, inhibition of NMT1 resulted in downregulation of late endosome/lysosomal adaptor, MAPK and mTOR activator 1 (LAMTOR1) myristoylation levels, which prevented LAMTOR1 from localizing itself to the lysosomal membrane, which in turn prevented mTORC1 activation, ultimately leading to slower growth and proliferation of cancer cells [32]. In addition, inhibition of NMT1 impairs lysosomal degradation, leading to blocked autophagic flux (Figure 1 (③)). However, the exact mechanism by which NMT1 and LAMTOR1 promote lysosomal degradation in cancer cells remains to be determined. Sun et al. [55] observed the same phenomenon in bladder cancer and also found that NMT1 knockdown not only prevented LAMTOR1 localization but also increased LAMTOR1 protein ubiquitination and decreased total LAMTOR1 protein levels [55]. These findings suggest that NMT1 affects lysosome-related functions through multiple aspects. As cancer cells are particularly sensitive to changes in the lysosome, however, blocking a single lysosomal metabolic function, for example, with chloroquine (which inhibits degradation) or mTOR inhibitor treatment, usually results in cellular adaptation [56]. Blocking NMT1-mediated myristoylation blocks both lysosomal catabolic and anabolic functions and means that targeting NMT1 is a promising anti-cancer strategy.

NMT1-mediated myristoylation is associated with activation of the Src oncogene; however, NMT1-mediated myristoylation may also have a role in preventing carcinogenesis. Uno et al. [57] provided evidence that myristoylation-deficient mutants cell lines of Fus1 (also known as TUSC2 tumour suppressor candidate 2, a tumour suppressor associated with lung cancer) lose tumour suppressor activity, and thus myristoylation may play a role in delaying lung carcinogenesis; however, it is unclear whether NMT1 or NMT2 is the catalyst in this process. In summary, drug research is necessary to identify NMT1-specific inhibitors for potential therapeutic use in lung and breast cancers.

### Endoplasmic reticulum stress

In breast and colon cancer cell models, small-molecule-mediated NMT inhibition induces ER stress, which subsequently leads to cell cycle arrest and apoptosis [58]. Deng et al. [59] postulated that a certain degree of NMT1 knockdown disrupts protein translation and processing in the ER and increases protein degradation and thus triggers ER stress. Both oxidative stress and ER stress can activate the c-Jun N-terminal kinase (JNK) pathway, leading to autophagy (Figure 1, ④); thus inhibiting breast cancer progression (Figure 1, ④).

Pharmacological stimulation to produce ER stress can impose an additional burden on cell survival, activating pro-apoptotic pathways and cell death [60]; therefore, several drugs that promote ER stress are currently used in cancer treatment [61]. However, these compounds ultimately increase the sensitivity or resistance of cancer cells to anti-cancer treatments. As both the proteasome and autophagy are vital in responding to ER stress by degrading unfolded proteins, simultaneous regulation of these pathways may be effective in increasing ER stress, and inhibitors targeting NMT1 provide such a unified approach [61].

### Lipid recoding

Evidence suggests an association of elevated fatty acids with ovarian cancer progression and metastasis [62,63]. Kim et al. [50] also observed that a high-fat diet (up to 60% fat) was associated with prostate cancer progression. A recent study revealed the relationship between lipid recoding by modulating myristoylation and metastasis of ovarian cancer cells [64]. The investigators found that the upregulation of acyl-CoA synthetase 1 (a major acyl-CoA synthetase in fatty acid metabolism) in highly metastatic ovarian cancer cells led to an increase in endogenous myristate acid and, as a consequence, the elevation of fatty acid oxidation. Increased myristate acid levels can enhance AMPK signalling in ovarian cancer cells because of myristoylation of the AMPK β-subunit (AMPKβ is specifically regulated by NMT1 rather than NMT2). Increased AMPKβ myristoylation is associated with AMPKα phosphorylation, which then leads to activation of fatty acid oxidation [64]. Furthermore, in ovarian cancer, Src activation increases cell survival and enhances drug resistance, while Src inhibition restores cell sensitivity to paclitaxel and cisplatin [65]. Thus, it is reasonable to assume that ovarian cancer cells evade immune surveillance and drug resistance by lipid recoding [65]. Given the converging role of NMT1 in AMPK and the Src pathway, for ovarian cancers prone to metastasis or drug resistance, the combination of NMT1 inhibitors and existing drugs may provide a new therapeutic avenue.

Notably, in addition to affecting tumour cells, many myristoylated proteins are also involved in immune surveillance process, which ultimately affect the tumour microenvironment. For example, myristoylation of TRAM in innate immunity localizes this protein to the plasma membrane as a prerequisite for lipopolysaccharide signalling [66]; whereas myristoylation of Lck during T-cell antigen receptor signalling affects the activation of CD3ζ, Zap70 and ERK, as well as the release of cytokines IFN-γ and IL-2 [67]; and myristoylation of the tumour suppressor Fus1 [57], which is associated with the Ca^2+^-myristoyl switch protein family, has been identified to balance mitochondrial changes during CD4+ T-cell activation [68]. These regulatory myristoylation suggest that other myristoylated proteins involved in signalling and protein localization under normal physiological conditions play roles in tumour development and growth; however, the presence of a direct relationship between NMT1 and these proteins remain unclear; and thus an in-depth study is warranted.

## NMT inhibitors

NMT1 expression and activity are altered in various types of cancer tissues. Furthermore, increasing evidence demonstrates that NMT1-mediated myristoylation is directly involved in oncogene signalling and cellular metabolic processes. As a consequence, selective inhibition of NMT1 function to control malignancies is a new approach in the field of anti-cancer drugs. Research to uncover the potential clinical applications of NMT inhibitors is summarized below.

The myristoyl coenzyme A analogue B13 and its derivative LCL204 were identified as small molecule inhibitors of NMT [50]. B13 prevents Src myristoylation and Src localization to the cytoplasmic membrane and attenuates Src-mediated oncogenic signalling. B13 has anti-invasive and anti-tumour effects on prostate [50] and bladder cancer cells with low toxic effects [55]. The use of these small molecule inhibitors provides a promising approach to inhibiting Src family kinase-mediated oncogenic activity [50]. The organopalladium compound dibenzylideneacetone dipalladium (Tris-DBA), is another inhibitor of human NMT synthesis, that blocks the kinase activity of NMT1 and decreases its expression. Tris-DBA shows potent anti-proliferative activity against melanoma cells by inhibiting several proliferation-related signalling pathway proteins including MAPK, Akt and STAT-3 [69]. However, the poor solubility of Tris-DBA impairs its effectiveness. Excitingly, a study synthesized Tris-DBA-Pd hyaluronic acid nanoparticles and demonstrated that this nanoparticle is an effective treatment against CD44-positive tumours such as melanoma [70]. Drug-like inhibitors IMP-366 (DDD85646) and IMP-1088 deliver complete and specific inhibition of N-myristoylation. In cancer cell lines, IMP-366 downregulates cell cycle regulatory proteins, and upregulates proteins involved in ER stress and the unfolded protein response [58]. IMP-1088, another inhibitor of both NMTs, completely inhibits N-myristoylation in a range of cell lines at low nanomolar concentrations (100 nM) [71].

Cases of chronic granulocytic leukaemia containing certain kinase mutations are resistant to tyrosine kinase inhibitor therapy. To overcome this resistance, investigators developed PCLX-001, which is a potent inhibitor in hematologic malignancies. PCLX-001 acts by attenuating Src myristoylation, basal Src levels and B-cell receptor downstream survival signals leading to apoptosis [33]. The results of a cancer cell line screen suggested the potential of PCLX-001 for wider application in treating leukaemia and myeloma, and certain solid tumours, such as breast and lung cancer. Subsequently, PCLX-001 effectively killed multiple breast cancer cell line subtypes, including breast and ductal carcinomas, in both *in vitro* and *in vivo* models [31]. Encouragingly, an open phase I clinical trial is currently underway to determine the maximum tolerated dose of PCLX-001 in treating B-cell lymphoma [6].

Antibody–drug conjugates (ADCs) are the fastest-growing class of drugs in the field of tumour therapy in recent years [72]. ADCs provide tumour-targeted therapy, and consist of a monoclonal antibody with a specific tumour-targeting effect, a small molecule cytotoxin with strong cell-killing power, and a linker connecting the two. The advantage of ADCs is that the small molecule cytotoxins effectively kill tumour cells without damaging or causing less damage to healthy tissue. A disadvantage of ADCs is that only a few cytotoxins are used, and each has a single mechanism of action [73]. NMT inhibitors, like IMP-1088, hold potential as future 'warheads' in ADCs, as tumour cell inhibition is high and effective concentrations are at the nanomolar level.

## Conclusions and perspectives

This review summarizes the current understanding of the role of NMT1 in tumour development. High NMT1 expression in various tumours may enable the use of NMT1 inhibitors to treat tumours. First, targeting the NMT1–Src axis offers a potential approach to inhibit tumour progression, especially in Src-driven tumours. In this situation, the inhibitor might selectively target the tumour, minimizing adverse effects in healthy tissues. In addition, growing evidence suggests that NMT1-mediated myristoylation plays a pivotal role in cancer cell metabolism (e.g. in lung and breast cancers) and may be particularly relevant to cancer metastasis and drug resistance. In the future, underlying mechanisms of sensitivity or resistance can be analysed in drug-resistant (or highly metastatic) and non-drug-resistant (or ground metastatic) cell lines through proteomic analysis of NMT1 substrate profiles and proteomic changes.

An initial framework has been formed from previous findings to explore the multiple functions of NMT1 in tumours. However, numerous challenges and uncertainties remain in targeting NMT1 as a clinical approach. First, specific substrates that target NMT1 have not been identified in detail. Recent studies have revealed that human N-terminal glycine NMT1 and two efficiently myristoylate specific lysine residues [74], which uncovers a previously unknown function of NMT1. Accordingly, identifying the substrate specificity of NMT1 requires further investigation. Second, NMT1 lacks specific inhibitors for basic cancer research. Although B13 and its derivative LCL204 [50], Tris DBA had an optimal inhibitory effect on NMT1 [69], have been discovered, these drugs are both inhibitors for a broad spectrum of NMT. There are no reports of NMT1/NMT2-selective inhibitors, probably because of the high sequence conservation between their catalytic domains [71]. Given their functional preferences, developing targeted inhibitors of NMT1 and NMT2 is required. Third, improving the efficacy of inhibitors can be continuously developed, such as combining nanomaterials or ADCs. Nonetheless, because of the many circumstances in which myristoylation is beneficial (e.g. myristoylation-dependent immune surveillance), caution is required when using an NMT1 inhibitor either as a single or combined therapy against cancers. Furthermore, the wide variety of NMT1 substrates introduces additional issues concerning NMT1 inhibition. NMT1-targeting strategies should be carefully designed according to each specific situation.

## Data Availability

Data sharing is not applicable to this article as no new data were created or analysed in this study.

## References

[1] Hanahan D. Hallmarks of cancer: new dimensions. Cancer Discov. 2022;12(1):31–46. 35022204 10.1158/2159-8290.CD-21-1059

[2] Lothrop AP, Torres MP, Fuchs SM. Deciphering post-translational modification codes. FEBS Lett. 2013;587(8):1247–1257. 23402885 10.1016/j.febslet.2013.01.047 PMC3888991

[3] Pan S, Chen R. Pathological implication of protein post-translational modifications in cancer. Mol Aspects Med. 2022;86:101097. 35400524 10.1016/j.mam.2022.101097 PMC9378605

[4] Li W, Li F, Zhang X, et al. Insights into the post-translational modification and its emerging role in shaping the tumor microenvironment. Signal Transduct Target Ther. 2021;6(1):422. 34924561 10.1038/s41392-021-00825-8 PMC8685280

[5] Ko PJ, Dixon SJ. Protein palmitoylation and cancer. EMBO Rep. 2018;19(10):e46666. 30232163 10.15252/embr.201846666 PMC6172454

[6] Fhu CW, Ali A. Protein lipidation by palmitoylation and myristoylation in cancer. Front Cell Dev Biol. 2021;9:673647. 34095144 10.3389/fcell.2021.673647 PMC8173174

[7] Wang B, Dai T, Sun W, et al. Protein N-myristoylation: functions and mechanisms in control of innate immunity. Cell Mol Immunol. 2021;18(4):878–888. 33731917 10.1038/s41423-021-00663-2 PMC7966921

[8] Farazi TA, Waksman G, Gordon JI. The biology and enzymology of protein N-myristoylation. J Biol Chem. 2001;276(43):39501–39504. 11527981 10.1074/jbc.R100042200

[9] Dian C, Perez-Dorado I, Riviere F, et al. High-resolution snapshots of human N-myristoyltransferase in action illuminate a mechanism promoting N-terminal Lys and Gly myristoylation. Nat Commun. 2020;11(1):1132. 32111831 10.1038/s41467-020-14847-3 PMC7048800

[10] Meinnel T, Dian C, Giglione C. Myristoylation, an ancient protein modification mirroring eukaryogenesis and evolution. Trends Biochem Sci. 2020;45(7):619–632. 32305250 10.1016/j.tibs.2020.03.007

[11] Tate EW, Bell AS, Rackham MD, et al. N-Myristoyltransferase as a potential drug target in malaria and leishmaniasis. Parasitology. 2014;141(1):37–49. 23611109 10.1017/S0031182013000450

[12] Wright MH, Heal WP, Mann DJ, et al. Protein myristoylation in health and disease. J Chem Biol. 2010;3(1):19–35. 19898886 10.1007/s12154-009-0032-8 PMC2816741

[13] Rajala RV, Datla RS, Moyana TN, et al. N-myristoyltransferase. Mol Cell Biochem. 2000;204(1–2):135–155. 10718634 10.1023/a:1007012622030

[14] Raju RV, Magnuson BA, Sharma RK. Mammalian myristoyl CoA: protein N-myristoyltransferase. Mol Cell Biochem. 1995;149–150(1):191–202. 10.1007/BF01076577 8569729

[15] Zha J, Weiler S, Oh KJ, et al. Posttranslational N-myristoylation of BID as a molecular switch for targeting mitochondria and apoptosis. Science. 2000;290(5497):1761–1765. 11099414 10.1126/science.290.5497.1761

[16] Utsumi T, Sakurai N, Nakano K, et al. C-terminal 15 kDa fragment of cytoskeletal actin is posttranslationally N-myristoylated upon caspase-mediated cleavage and targeted to mitochondria. FEBS Lett. 2003;539(1–3):37–44. 12650923 10.1016/s0014-5793(03)00180-7

[17] Zhang J, Zeng Y, Xing Y, et al. Myristoylation-mediated phase separation of EZH2 compartmentalizes STAT3 to promote lung cancer growth. Cancer Lett. 2021;516:84–98. 34102285 10.1016/j.canlet.2021.05.035

[18] Maurer-Stroh S, Eisenhaber F. Myristoylation of viral and bacterial proteins. Trends Microbiol. 2004;12(4):178–185. 15051068 10.1016/j.tim.2004.02.006

[19] Martin DD, Vilas GL, Prescher JA, et al. Rapid detection, discovery, and identification of post-translationally myristoylated proteins during apoptosis using a bio-orthogonal azidomyristate analog. FASEB J. 2008;22(3):797–806. 17932026 10.1096/fj.07-9198com PMC2865240

[20] Udenwobele DI, Su RC, Good SV, et al. Myristoylation: an important protein modification in the immune response. Front Immunol. 2017;8:751. 28713376 10.3389/fimmu.2017.00751 PMC5492501

[21] Selvakumar P, Lakshmikuttyamma A, Shrivastav A, et al. Potential role of N-myristoyltransferase in cancer. Prog Lipid Res. 2007;46(1):1–36. 16846646 10.1016/j.plipres.2006.05.002

[22] Broncel M, Dominicus C, Vigetti L, et al. Profiling of myristoylation in *Toxoplasma gondii* reveals an N-myristoylated protein important for host cell penetration. Elife. 2020;9:e57861. 32618271 10.7554/eLife.57861 PMC7373427

[23] Takamune N, Gota K, Misumi S, et al. HIV-1 production is specifically associated with human NMT1 long form in human NMT isozymes. Microbes Infect. 2008;10(2):143–150. 18248763 10.1016/j.micinf.2007.10.015

[24] Duronio RJ, Towler DA, Heuckeroth RO, et al. Disruption of the yeast N-myristoyl transferase gene causes recessive lethality. Science. 1989;243(4892):796–800. 2644694 10.1126/science.2644694

[25] Duronio RJ, Rudnick DA, Johnson RL, et al. Myristic acid auxotrophy caused by mutation of *S. cerevisiae* myristoyl-CoA:protein N-myristoyltransferase. J Cell Biol. 1991;113(6):1313–1330. 2045414 10.1083/jcb.113.6.1313 PMC2289034

[26] Yang SH, Shrivastav A, Kosinski C, et al. N-myristoyltransferase 1 is essential in early mouse development. J Biol Chem. 2005;280(19):18990–18995. 15753093 10.1074/jbc.M412917200

[27] Corbic Ramljak I, Stanger J, Real-Hohn A, et al. Cellular N-myristoyltransferases play a crucial picornavirus genus-specific role in viral assembly, virion maturation, and infectivity. PLoS Pathog. 2018;14(8):e1007203. 30080883 10.1371/journal.ppat.1007203 PMC6089459

[28] Giang DK, Cravatt BF. A second mammalian N-myristoyltransferase. J Biol Chem. 1998;273(12):6595–6598. 9506952 10.1074/jbc.273.12.6595

[29] Ducker CE, Upson JJ, French KJ, et al. Two N-myristoyltransferase isozymes play unique roles in protein myristoylation, proliferation, and apoptosis. Mol Cancer Res. 2005;3(8):463–476. 16123142 10.1158/1541-7786.MCR-05-0037 PMC2908404

[30] Chen L, Ling B, Alcorn J, et al. Quantitative analysis of the expression of human N-myristoyltransferase 1 (hNMT-1) in cancers. Open Biomark J. 2009;2(1):6–10.

[31] Mackey JR, Lai J, Chauhan U, et al. N-myristoyltransferase proteins in breast cancer: prognostic relevance and validation as a new drug target. Breast Cancer Res Treat. 2021;186(1):79–87. 33398478 10.1007/s10549-020-06037-y PMC7940342

[32] Chen YC, Navarrete MS, Wang Y, et al. N-myristoyltransferase-1 is necessary for lysosomal degradation and mTORC1 activation in cancer cells. Sci Rep. 2020;10(1):11952. 32686708 10.1038/s41598-020-68615-w PMC7371688

[33] Beauchamp E, Yap MC, Iyer A, et al. Targeting N-myristoylation for therapy of B-cell lymphomas. Nat Commun. 2020;11(1):5348. 33093447 10.1038/s41467-020-18998-1 PMC7582192

[34] Magnuson BA, Raju RV, Moyana TN, et al. Increased N-myristoyltransferase activity observed in rat and human colonic tumors. J Natl Cancer Inst. 1995;87(21):1630–1635. 7563206 10.1093/jnci/87.21.1630

[35] Raju RV, Moyana TN, Sharma RK. N-Myristoyltransferase overexpression in human colorectal adenocarcinomas. Exp Cell Res. 1997;235(1):145–154. 9281363 10.1006/excr.1997.3679

[36] Felsted RL, Glover CJ, Hartman K. Protein N-myristoylation as a chemotherapeutic target for cancer. J Natl Cancer Inst. 1995;87(21):1571–1573. 7563194 10.1093/jnci/87.21.1571

[37] Shrivastav A, Varma S, Saxena A, et al. N-myristoyltransferase: a potential novel diagnostic marker for colon cancer. J Transl Med. 2007;5:58. 18021392 10.1186/1479-5876-5-58 PMC2203986

[38] Clegg RA, Gordge PC, Miller WR. Expression of enzymes of covalent protein modification during regulated and dysregulated proliferation of mammary epithelial cells PKA, PKC and NMT. Adv Enzyme Regul. 1999;39:175–203. 10470373 10.1016/s0065-2571(98)00011-9

[39] Shrivastav A, Varma S, Senger A, et al. Overexpression of Akt/PKB modulates N-myristoyltransferase activity in cancer cells. J Pathol. 2009;218(3):391–398. 19360752 10.1002/path.2550

[40] Liang J, Xu ZX, Ding Z, et al. Myristoylation confers noncanonical AMPK functions in autophagy selectivity and mitochondrial surveillance. Nat Commun. 2015;6:7926. 26272043 10.1038/ncomms8926

[41] Zhu G, Wang F, Li H, et al. N-Myristoylation by NMT1 is POTEE-dependent to stimulate liver tumorigenesis via differentially regulating ubiquitination of targets. Front Oncol. 2021;11:681366. 34136404 10.3389/fonc.2021.681366 PMC8201403

[42] Shrivastav A, Sharma A, Bajaj G, et al. Elevated N-myristoyltransferase activity and expression in oral squamous cell carcinoma. Oncol Rep. 2007;18(1):93–97. 17549352

[43] Lu Y, Selvakumar P, Ali K, et al. Expression of N-myristoyltransferase in human brain tumors. Neurochem Res. 2005;30(1):9–13. 15756927 10.1007/s11064-004-9680-9

[44] Rajala RVS. Increased expression of N-myristoyltransferase in gallbladder carcinomas. Am Cancer Soc. 2000;88(9):1992–1999. 10813869

[45] Phelan JD, Young RM, Webster DE, et al. A multiprotein supercomplex controlling oncogenic signalling in lymphoma. Nature. 2018;560(7718):387–391. 29925955 10.1038/s41586-018-0290-0 PMC6201842

[46] Caner A, Asik E, Ozpolat B. SRC signaling in cancer and tumor microenvironment. Adv Exp Med Biol. 2021;1270:57–71. 33123993 10.1007/978-3-030-47189-7_4

[47] Rajala RV, Dehm S, Bi X, et al. Expression of N-myristoyltransferase inhibitor protein and its relationship to c-Src levels in human colon cancer cell lines. Biochem Biophys Res Commun. 2000;273(3):1116–1120. 10891381 10.1006/bbrc.2000.3066

[48] Shoji S, Kurosawa T, Inoue H, et al. Human cellular Src gene product: identification of the myristoylated pp60c-src and blockage of its myristoyl acylation with N-fatty acyl compounds resulted in the suppression of colony formation. Biochem Biophys Res Commun. 1990;173(3):894–901. 2268350 10.1016/s0006-291x(05)80870-8

[49] Patwardhan P, Resh MD. Myristoylation and membrane binding regulate c-Src stability and kinase activity. Mol Cell Biol. 2010;30(17):4094–4107. 20584982 10.1128/MCB.00246-10 PMC2937550

[50] Kim S, Alsaidan OA, Goodwin O, et al. Blocking myristoylation of Src inhibits its kinase activity and suppresses prostate cancer progression. Cancer Res. 2017;77(24):6950–6962. 29038344 10.1158/0008-5472.CAN-17-0981 PMC5732839

[51] Bagrodia S, Taylor SJ, Shalloway D. Myristylation is required for Tyr-527 dephosphorylation and activation of pp60c-src in mitosis. Mol Cell Biol. 1993;13(3):1464–1470. 7680096 10.1128/mcb.13.3.1464-1470.1993 PMC359457

[52] Araujo JC, Trudel GC, Saad F, et al. Docetaxel and dasatinib or placebo in men with metastatic castration-resistant prostate cancer (READY): a randomised, double-blind phase 3 trial. Lancet Oncol. 2013;14(13):1307–1316. 24211163 10.1016/S1470-2045(13)70479-0 PMC5478530

[53] Lawrence RE, Zoncu R. The lysosome as a cellular centre for signalling, metabolism and quality control. Nat Cell Biol. 2019;21(2):133–142. 30602725 10.1038/s41556-018-0244-7

[54] Xu H, Ren D. Lysosomal physiology. Annu Rev Physiol. 2015;77:57–80. 25668017 10.1146/annurev-physiol-021014-071649 PMC4524569

[55] Sun Y, Guan Z, Sheng Q, et al. N-myristoyltransferase-1 deficiency blocks myristoylation of LAMTOR1 and inhibits bladder cancer progression. Cancer Lett. 2022;529:126–138. 34999170 10.1016/j.canlet.2022.01.001

[56] Rebecca VW, Nicastri MC, McLaughlin N, et al. A unified approach to targeting the lysosome's degradative and growth signaling roles. Cancer Discov. 2017;7(11):1266–1283. 28899863 10.1158/2159-8290.CD-17-0741 PMC5833978

[57] Uno F, Sasaki J, Nishizaki M, et al. Myristoylation of the fus1 protein is required for tumor suppression in human lung cancer cells. Cancer Res. 2004;64(9):2969–2976. 15126327 10.1158/0008-5472.can-03-3702

[58] Thinon E, Morales-Sanfrutos J, Mann DJ, et al. N-Myristoyltransferase inhibition induces ER-Stress, cell cycle arrest, and apoptosis in cancer cells. ACS Chem Biol. 2016;11(8):2165–2176. 27267252 10.1021/acschembio.6b00371 PMC5077176

[59] Deng L, Gao X, Liu B, et al. NMT1 inhibition modulates breast cancer progression through stress-triggered JNK pathway. Cell Death Dis. 2018;9(12):1143. 30446635 10.1038/s41419-018-1201-x PMC6240078

[60] Urra H, Dufey E, Avril T, et al. Endoplasmic reticulum stress and the hallmarks of cancer. Trends Cancer. 2016;2(5):252–262. 28741511 10.1016/j.trecan.2016.03.007

[61] Nagelkerke A, Bussink J, Sweep FC, et al. The unfolded protein response as a target for cancer therapy. Biochim Biophys Acta. 2014;1846(2):277–284. 25069067 10.1016/j.bbcan.2014.07.006

[62] Pradeep S, Kim SW, Wu SY, et al. Hematogenous metastasis of ovarian cancer: rethinking mode of spread. Cancer Cell. 2014;26(1):77–91. 25026212 10.1016/j.ccr.2014.05.002 PMC4100212

[63] Tucker SL, Gharpure K, Herbrich SM, et al. Molecular biomarkers of residual disease after surgical debulking of high-grade serous ovarian cancer. Clin Cancer Res. 2014;20(12):3280–3288. 24756370 10.1158/1078-0432.CCR-14-0445 PMC4062703

[64] Zhang Q, Zhou W, Yu S, et al. Metabolic reprogramming of ovarian cancer involves ACSL1-mediated metastasis stimulation through upregulated protein myristoylation. Oncogene. 2021;40(1):97–111. 33082557 10.1038/s41388-020-01516-4

[65] Pengetnze Y, Steed M, Roby KF, et al. Src tyrosine kinase promotes survival and resistance to chemotherapeutics in a mouse ovarian cancer cell line. Biochem Biophys Res Commun. 2003;309(2):377–383. 12951060 10.1016/j.bbrc.2003.08.012

[66] Rowe DC, Latz MA, Monks E, et al. The myristoylation of TRIF-related adaptor molecule is essential for toll-like receptor 4 signal transduction. Proc Natl Acad Sci U S A. 2006;103(16):6299–6304. 16603631 10.1073/pnas.0510041103 PMC1458872

[67] Rampoldi F, Bonrouhi M, Boehm ME, et al. Immunosuppression and aberrant T cell development in the absence of N-myristoylation. J Immunol. 2015;195(9):4228–4243. 26423150 10.4049/jimmunol.1500622

[68] Uzhachenko R, Ivanov SV, Yarbrough WG, et al. Fus1/Tusc2 is a novel regulator of mitochondrial calcium handling, Ca^2+^-coupled mitochondrial processes, and Ca^2+^-dependent NFAT and NF-kappaB pathways in CD4+ T cells. Antioxid Redox Signal. 2014;20(10):1533–1547. 24328503 10.1089/ars.2013.5437 PMC3942676

[69] Bhandarkar SS, Bromberg J, Carrillo C, et al. Tris (dibenzylideneacetone) dipalladium, a N-myristoyltransferase-1 inhibitor, is effective against melanoma growth in vitro and in vivo. Clin Cancer Res. 2008;14(18):5743–5748. 18794083 10.1158/1078-0432.CCR-08-0405 PMC4423743

[70] Elsey J, Bubley JA, Zhu L, et al. Palladium based nanoparticles for the treatment of advanced melanoma. Sci Rep. 2019;9(1):3255. 30824801 10.1038/s41598-019-40258-6 PMC6397149

[71] Kallemeijn WW, Lueg GA, Faronato M, et al. Validation and invalidation of chemical probes for the human N-myristoyltransferases. Cell Chem Biol. 2019;26(6):892–900.e4. 31006618 10.1016/j.chembiol.2019.03.006 PMC6593224

[72] Hafeez U, Parakh S, Gan HK, et al. Antibody–drug conjugates for cancer therapy. Molecules. 2020;25(20):4764. 33081383 10.3390/molecules25204764 PMC7587605

[73] Desai A, Abdayem P, Adjei AA, et al. Antibody–drug conjugates: a promising novel therapeutic approach in lung cancer. Lung Cancer. 2022;163:96–106. 34942494 10.1016/j.lungcan.2021.12.002

[74] Kosciuk T, Price IR, Zhang X, et al. NMT1 and NMT2 are lysine myristoyltransferases regulating the ARF6 GTPase cycle. Nat Commun. 2020;11(1):1067. 32103017 10.1038/s41467-020-14893-x PMC7044312

